# Enhancing the Silanization Reaction of the Silica-Silane System by Different Amines in Model and Practical Silica-Filled Natural Rubber Compounds

**DOI:** 10.3390/polym10060584

**Published:** 2018-05-27

**Authors:** C. Hayichelaeh, L.A.E.M. Reuvekamp, W.K. Dierkes, A. Blume, J.W.M. Noordermeer, K. Sahakaro

**Affiliations:** 1 Department of Rubber Technology and Polymer Science, Faculty of Science and Technology, Prince of Songkla University, Pattani 94000, Thailand; c.hayichelaeh@utwente.nl; 2 Elastomer Technology and Engineering, Department of Mechanics of Solids, Surfaces and Systems (MS3), Faculty of Engineering Technology, University of Twente, P.O. BOX 217, 7500AE Enschede, The Netherlands; l.a.e.m.reuvekamp@utwente.nl (L.A.E.M.R.); w.k.dierkes@utwente.nl (W.K.D.); a.blume@utwente.nl (A.B.); 3 Apollo Tyres Global R&D B.V., Colosseum 2, 7521PT Enschede, The Netherlands

**Keywords:** silanization, amine, silica, silane, model compound

## Abstract

Diphenyl guanidine (DPG) is an essential ingredient in silica-reinforced rubber compounds for low rolling resistance tires, as it not only acts as a secondary accelerator, but also as a catalyst for the silanization reaction. However, because of concern over the toxicity of DPG that liberates aniline during high-temperature processing, safe alternatives are required. The present work studies several amines as potential alternatives for DPG. Different amines (i.e., hexylamine, decylamine, octadecylamine, cyclohexylamine, dicyclohexylamine, and quinuclidine) are investigated in a model system, as well as in a practical rubber compound by taking the ones with DPG and without amine as references. The kinetics of the silanization reaction of the silica/silane mixtures are evaluated using model compounds. The mixtures with amines show up to 3.7 times higher rate constants of the primary silanization reaction compared to the compound without amine. Linear aliphatic amines promote the rate constant of the primary silanization reaction to a greater extent compared to amines with a cyclic structure. The amines with short-alkyl chains that provide better accessibility towards the silica surface, enhance the primary silanization reaction more than the ones with long-alkyl chains. The different amines have no significant influence on the rate constant of the secondary silanization reaction. The amine types that give a higher primary silanization reaction rate constant show a lower flocculation rate in the practical compounds. For the systems with a bit lower primary silanization reaction rate, but higher extent of shielding or physical adsorption that still promotes higher interfacial compatibility between the elastomer and the filler surface, the rubber compounds show a lower Payne effect which would indicate lower filler-filler interaction. However, the flocculation rate constant remained high.

## 1. Introduction

Driven by the need for more environmentally friendly and regulated products, low rolling resistance tires have been developed based on utilization of silica/silane technology for rubber reinforcement [1]. Highly dispersible silica fillers in combination with a silane coupling agent need to be mixed into the rubber under optimized mixing conditions [2,3] using a suitable formulation [4] to ensure the occurrence of the silanization reaction that leads to bonding of the silane moiety onto the silica surface. As a consequence of the silanization reaction during mixing between the alkoxy groups of the silane molecules and silanol groups on the silica surface, and the subsequent coupling reaction between the bound silane moieties and elastomer molecules during the curing process, chemical linkages between the silica and the elastomer molecules are created forming the basis for low rolling resistance tire treads. This filler-elastomer network, in combination with elastomer crosslinks, leads to low hysteresis of the silica-filled rubber vulcanizates.

The extent of the silanization reaction is a crucial factor to be optimized in order to maximize the end-use properties of the rubber vulcanizates. The functional groups of the silane molecules play an important role in the silica/rubber system, affecting filler-to-elastomer interfacial adhesion [5]. The silanization reaction enhances silica-elastomer interactions, reduces filler-filler interactions, and lowers the compound viscosity, which is beneficial for both processing and vulcanizate properties. The most commonly used silane coupling agent is bis(3-triethoxysilylpropyl)-tetrasulfide (TESPT) with alkoxy- and sulfur-functional groups [2,5]. For silica/TESPT-filled rubber compounds, the optimum discharge temperature of mixing has a strong influence on the extent of the silanization reaction [2,3], where the recommended discharge temperature for silica-reinforced natural rubber (NR) compounds is in the range of 135–150 °C [3]. The presence of an amine-based secondary accelerator such as diphenyl guanidine (DPG) in the silica-reinforced rubber compounds plays an additional role as a catalyst booster for the silanization reaction [6,7]. However, due to the potential liberation of toxic aniline by DPG [8] during high processing temperatures (i.e., during mixing and vulcanization), and consequent concerns over its safety, safe alternatives for DPG are required.

Indirect indications for the extent of the silanization reaction in practical rubber compounds, which relate to filler-elastomer interactions, include chemically bound rubber content [9], the Payne effect [10], flocculation rate constant [11], and heat capacity increment [12,13]. In order to obtain an in-depth insight into the silanization reaction, a study on the kinetics of the silica-silane system is needed. Based on the silanization study by Görl et al. [14] using a model system, it was concluded that the reaction proceeds in two steps. The primary reaction involves the first out of three ethoxy-groups on one TESPT silicon atom. The secondary follow-up reaction involves the second and potentially third ethoxy-groups. The primary reaction is about 10–20 times faster than the secondary reaction. The reaction can occur via two pathways: direct condensation and hydrolysis prior to condensation, while the secondary reaction encompasses condensation between adjacent alkoxy groups of TESPT already bound to the silica surface. A recent report by Blume and Thibault-Starzyk [15] on the silica-silane reaction mechanism characterized by in situ infrared spectroscopy revealed that only the isolated and geminal silanol groups were involved in the reaction. Only 25% of the Si-OH groups is estimated to react with silanes due to the accessibility of these silanol groups to incoming silane molecules. Based on molecular modeling using a (3-mercaptopropyl)triethoxysilane (Si263) silane coupling agent, two molecules can only react to two silanol groups with a distance higher than 0.4 nm, so the number of silanes grafted on the silica surface is limited. An increase in the hydrophobation of the silica surface is possible through the use of small molecules such as alcohols and amines, or silanes with high shielding potential [15,16]. The reactions are accelerated by an increase in temperature and by acidic or alkaline conditions. A study on the kinetics of different silanes with varying functional groups in model systems revealed that, although only the alkoxy group reacted with the silanol group of the silica, the chemical structure of the whole silane influenced the rate of the reaction [17]. Mihara [18] studied the silanization kinetics of silica/silane in a model Olefin system in the presence of different amines with various acid dissociation constant (pKa) values ranging from 1.30 to 11.5. It was shown that for pKa ≥ 6.5, the rate constant increased and the activation energy decreased. Quinuclidine and 3-quinuclidinol with respective pKa values of 11.5 and 10.1 gave high silanization rates and were tested in a tire tread compound in comparison to DPG with a pKa of 10.1.

The present work explores the potential of amines having different structures with pKa’s in the range of 10–12 as alternatives for DPG. Different amine types (i.e., hexylamine (HEX), decylamine (DEC), octadecylamine (OCT), cyclohexylamine (CYC), dicyclohexylamine (DIC), and quinuclidine (QUI)) are used while taking silica/silane systems with DPG and without amine as references. The rate constants of the primary and secondary silanization reactions are determined based on a study in a model system. Additionally, the interfacial compatibility of practical silica-reinforced NR compounds with those amines is assessed.

## 2. Experimental

### 2.1. Materials

Highly dispersible silica ULTRASIL 7005 (Evonik, Marl, Germany), characterized by BET (Brunauer-Emmett-Teller) - and CTAB (Cetyl-Trimethyl-AmmoniumBromide)-specific surface areas of 180 and 171 m^2^/g, respectively, was used in this study. Diphenyl guanidine was obtained from Flexys, Belgium, and the other amines as detailed in Table 1 were from Sigma–Aldrich Chemie, Germany. Dimethyl sulfoxide (DMSO) was supplied by Sigma–Aldrich Chemie, Germany. The chemicals for the model compound study were anhydrous-grade decane (Sigma–Aldrich Chemie, Steinheim Germany), bis-(3-triethoxysilyl-propyl)tetrasulfide (TESPT) (Evonik, Germany), and diethyleneglycol monobutylether (Sigma–Aldrich Chemie, Germany). For compound preparation, the NR used was ribbed smoked sheet (RSS#3), locally produced in Pattani, Thailand. Other rubber compounding ingredients were treated distillate aromatic extract (TDAE) oil (Vivatec 500, Hansen & Rosenthal, Hamburg, Germany), 2,2,4-TriMethyl-1,2-dihydroQuinoline (TMQ) (Flexys, Antwerp, Belgium), zinc oxide and stearic acid (both from Sigma–Aldrich Chemie).

### 2.2. Preparation and Characterization of the Aniline Liberated from DPG

For preparation, 0.2 g of DPG was added to a vial containing 5 mL of DMSO. The vials containing the mixtures were then heated at different temperatures (i.e., 130, 150, and 170 °C) for different times. After reaching the required time, the mixtures were quenched in an ice bath. Then, the concentration of aniline was determined by gas chromatography (GC) using a GC-2010 plus (Shimadzu, Kyoto, Japan). The chromatographic conditions are given in Table 2.

### 2.3. Preparation and Characterization of the Model Reaction Mixtures

Silica was first heated in an oven at 100 °C for 2 h prior to being used. Then, 0.5 g of silica was added into a vial before adding 0.94 mmol of TESPT and 0.30 mmol of the respective amines. Reactions with DPG and without amine were also prepared as references. Thereafter, 4 mL of *n*-decane was added. The vials containing the reaction mixtures were then flushed with inert nitrogen gas, before being immersed into an oil bath at 135 ± 2 °C for different reaction times under continuous stirring. After reaching the required time, the reactions were quenched by cooling in an ice bath. All physical interactions on the silica surfaces were removed by adding 2 mL of diethyleneglycol monobutylether to the reaction mixtures, following the method reported by Blume [17]. This step also desorbed ethanol from the silica surface, generated by the silanization reaction. The resulting mixtures were subsequently filtered and then the concentration of ethanol (EtOH) was determined by GC as detailed in Section 2.2.

### 2.4. Preparation of Rubber Compounds without Curatives

Rubber compounds were prepared in an internal mixer (Brabender Plasticorder 350S, Duisburg, Germany) with mixing conditions of fill factor 0.7, initial mixer temperature setting of 100 °C, and rotor speed of 60 rpm. The rubber formulation and mixing procedure are shown in Table 3 and Table 4, respectively. The discharge temperature of all mixes having different amine types were in the range of 131–135 °C. The rubber compounds were tested for the properties that can be related to filler-elastomer and filler-filler interactions: Payne effect, change in heat capacity, and flocculation rate constant.

### 2.5. Characterization of Filler-Elastomer and Filler-Filler Interactions in Uncured Rubber Compounds

*Payne effect:* The storage shear moduli (G’) of the rubber compounds were evaluated using a rubber process analyzer (RPA) (Alpha Technologies, Akron, OH, USA) at a temperature of 100 °C, frequency 0.5 Hz, and varying strains in the range of 0.28–100%. The Payne effect was calculated from the difference in storage shear moduli at low strain (0.56%) and high strain (100%) (i.e., G’(0.56%)–G’(100%)).

*Thermal behavior*: The glass transition temperature (*T*_g_) and heat capacity increment (*ΔC_p_*) at *T*_g_ of the rubber compounds were analyzed using differential scanning calorimetry (DSC 214 Polyma, Netzsch, Selb, Germany). The samples were weighed into standard aluminum pans and analyzed in the temperature range of −100 to +30 °C at a heating rate of 10 °C/min under nitrogen atmosphere. The heat history of the materials was removed in the first heating scan, and the data from the second heating scan were evaluated for changes in heat flow.

*Flocculation rate constant*: The flocculation rate constant (*K*) of the uncured silica-reinforced NR compounds was studied using the RPA at 100 °C, strain 0.56%, frequency 1.00 Hz, and test time of 12 min. The storage shear moduli at different times were recorded and the *K* calculated following Equations (1) and (2) [11]:

(1) $$x=\frac{s{}^{\prime }(t)-s{}^{\prime }(1)}{s{}^{\prime }(\infty )-s{}^{\prime }(1)}$$

where *x* is the degree of flocculation, *s′(t)* is the storage modulus at 0.56% strain at test time *t*, *s′(1)* is the storage modulus after preheating for 1 min, and *s′(∞)* is the storage modulus after heating for 12 min.

(2) $$K[\min^{-1}]=\frac{\ln(1-x_{1})-\ln(1-x_{2})}{t_{2}-t_{1}}$$

where *x_1_* and *x_2_* are the degree of flocculation at different heating times (i.e., *t_1_* and *t_2_*, respectively).

## 3. Results and Discussion

### 3.1. Liberated Aniline from DPG

**Figure 1:**
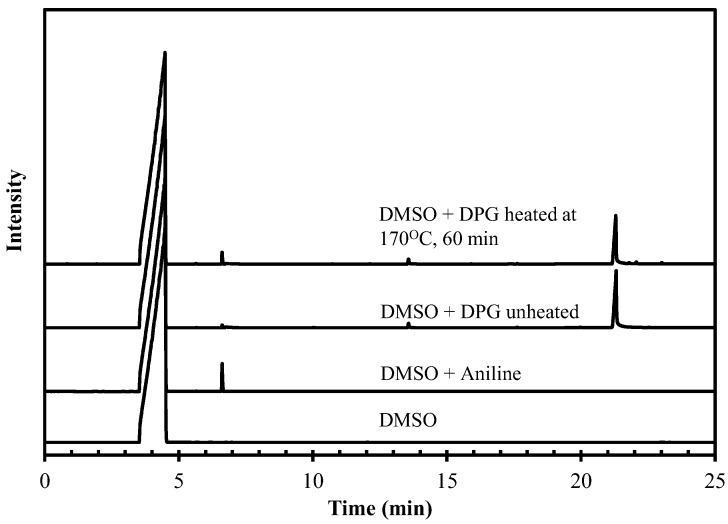
GC results showing aniline liberation from DPG. DMSO = dimethyl sulfoxide.

**Figure 2:**
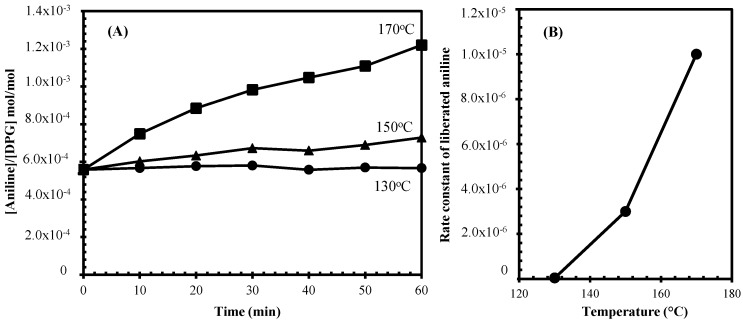
Concentration of aniline under heating at 130, 150, and 170 °C with different times (**A**) and the rate constant of aniline liberation (**B**).

Release of aniline from the decomposition of DPG at high temperature is illustrated in Figure 1 based on GC analysis. Aniline showed its peak at 6.5 min, while DPG appeared at 21 min. An integrated peak area was related to a concentration of each corresponding substance. Figure 2 shows that the concentration of aniline generated is dependent on temperature and a substantial amount of aniline can readily be observed above the temperature of 130 °C. The rate of liberated aniline rose with increasing temperature. For the silica-silane-rubber mixing that required a high temperature for the silanization, it was therefore unavoidable for aniline to be generated from DPG, and so a safe alternative is needed.

### 3.2. Silanization Kinetics in Model Olefin Systems

**Figure 3:**
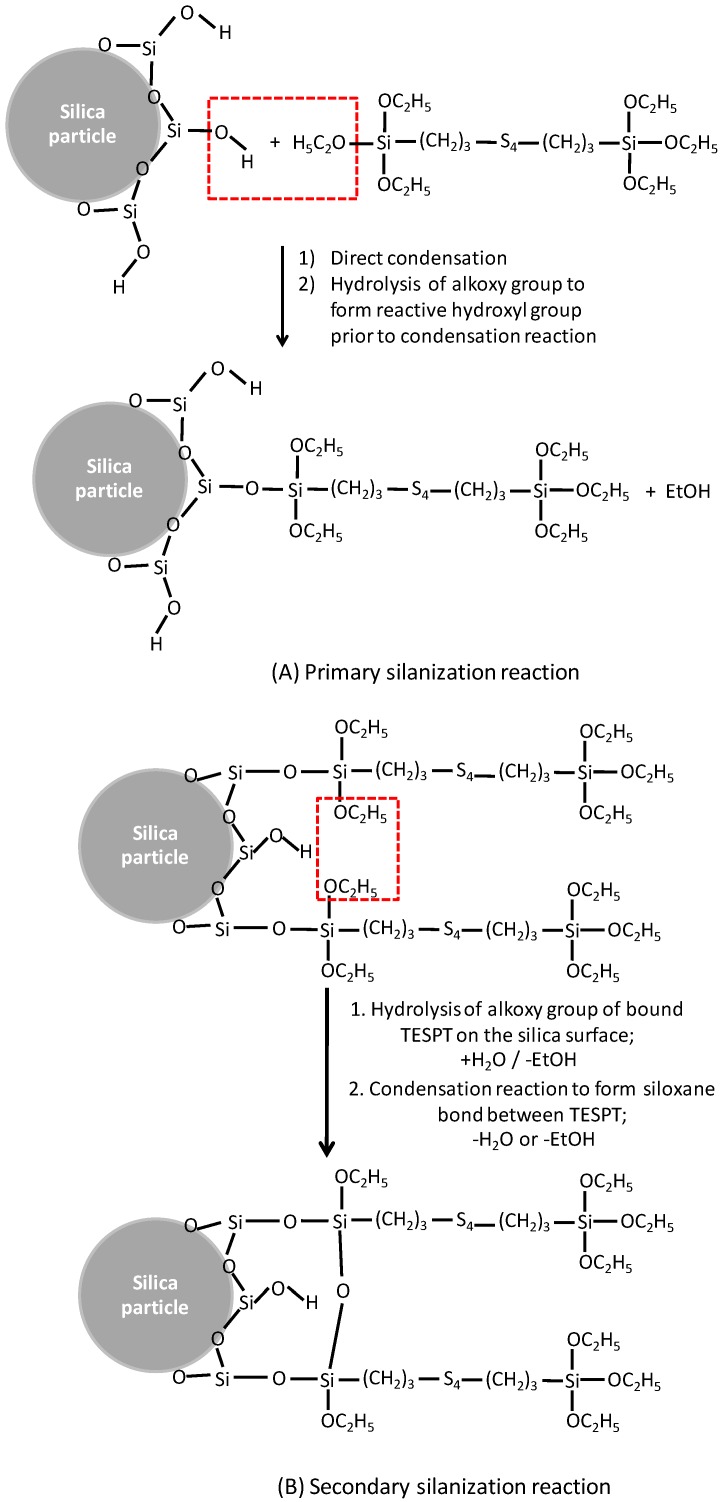
The primary and secondary silanization reactions in a silica/TESPT system (adapted from Reference [14]).

The success of TESPT as a silane coupling agent in silica/rubber systems was due to two main chemical reactions: silanization of the silica and coupling towards the elastomers that finally lead to chemical bridges between the silica surfaces and elastomer molecules. As stated before, the silanization reaction is divided into a primary and secondary reaction (Figure 3). The primary reaction can proceed via two pathways: either by direct condensation between the silanol groups of the silica surface and one alkoxy group of TESPT, or by hydrolysis of the alkoxy groups of TESPT to form reactive hydroxyl groups prior to the condensation reaction [14]. Both pathways release EtOH as byproduct. The secondary reaction often occurs between adjacent TESPT molecules on the filler surface, also releasing ethanol. 

**Figure 4:**
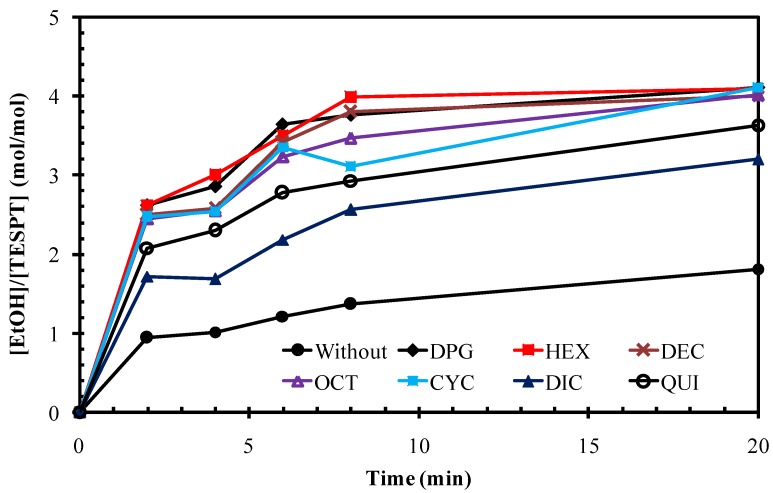
Ethanol released during the silanization reaction of silica/silane mixtures with different amine types.

**Figure 5:**
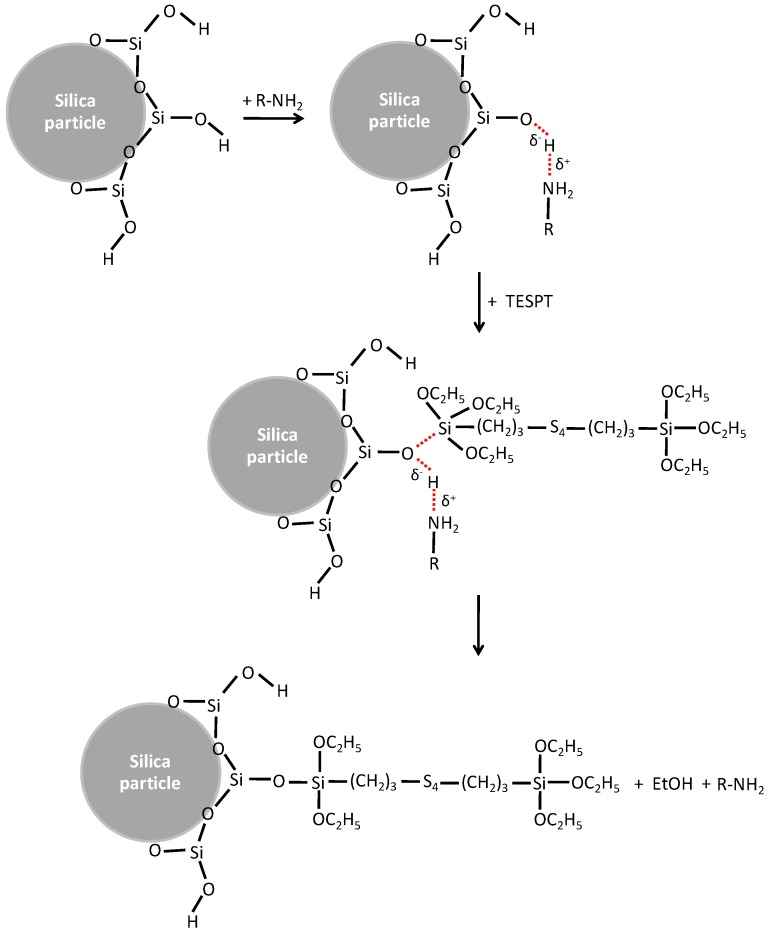
Interaction of amine with the silanol group and its contribution to the silanization (adapted from Reference [21]).

The EtOH released from the silanization reaction of the silica/silane system in the *n*-decane model compounds calculated in relation to the concentration of TESPT used is shown in Figure 4. The concentration of released EtOH increased with increasing reaction time. In the presence of amines, the ethanol concentration rose sharply within 10 min to exceed 2 moles per mole of TESPT due to the primary reaction, and thereafter rose more slowly. The silica/silane systems with all amine types showed significantly higher concentrations of EtOH than the one without amine due to the enhanced silanization reaction catalyzed by the alkaline amines. Hydrolysis of the silane can readily occur under basic conditions [19]. Thus, amine provides a catalytic effect on the hydrolysis of alkoxy groups in the silane molecules to form reactive hydroxyl moieties prior to the condensation reaction [19,20]. In the presence of amine and water, a hydroxyl ion (OH^−^) was formed, and EtOH was released from the TESPT molecules via a pentacoordinate intermediate, forming Si-OH [19,20]. Subsequently, the amines catalyzed the condensation reaction between the Si-OH on the silica surface and the alkoxy groups in the TESPT molecules [21,22,23]. The catalyzed condensation reaction occured after the amine entered into interaction with the silanol group on the silica surface via hydrogen bonding, enhancing more nucleophilicity on the silica surface. Thus, the silica surface could interact easier with the silicon atom in the TESPT molecule that led to the condensation reaction [21]. This role of amine as a catalyst for the silanization reaction is depicted in Figure 5.

In case of the primary silanization reaction, 1 mol of TESPT which reacted with silanol groups on the silica surface released 2 mols of EtOH as byproduct. The rate constant of the silanization reaction depended on the TESPT concentration, which can be described by a first order rate law. Based on the concentration of TESPT and released EtOH, the rate of the silanization reaction can be calculated using Equation (3) as shown below [14].

(3) $$-\frac{d[TESPT]}{dt}=k_{a}[TESPT]=\frac{1}{2}\frac{d[EtOH]}{dt}$$

where [*TESPT*] is concentration of TESPT; [*EtOH*] is a concentration of EtOH; *k_a_* is rate constant of primary silanization reaction, and *t* is time.

**Figure 6:**
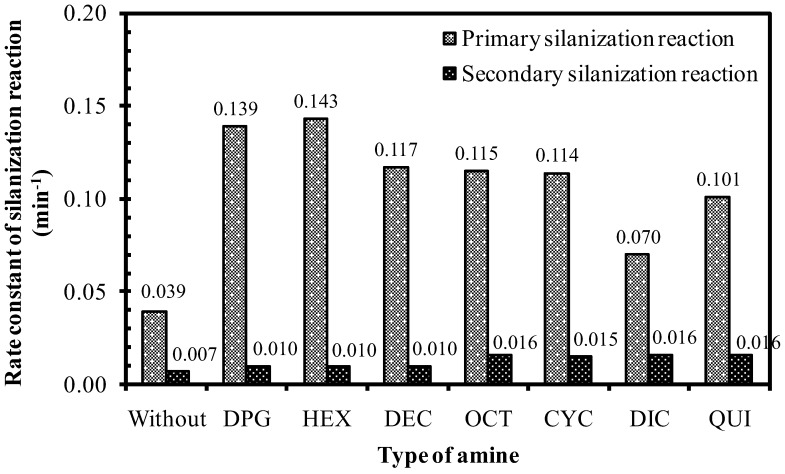
Rate constant of the silanization reaction of silica/silane mixtures with different amine types.

Based on Equation (3) and the model reaction procedure as laid out in Section 2.3, the rate constant of the primary silanization reaction can be calculated in the usual manner by plotting ln [TESPT]_t_ − ln [TESPT]_0_ against time *t*; where [TESPT]_t_ is the TESPT concentration at time *t* and [TESPT]_0_ is the initial TESPT concentration. The slope of the plot is taken as the reaction rate constant. Figure 6 shows the rate constants of the primary silanization reaction of silica/TESPT with different amine types in comparison with the reference without amine. It shows that the reaction mixture of silica/TESPT without amine displayed the lowest rate constant of the primary silanization reaction, equal to 0.039 min^−1^. The reference with DPG had a primary silanization reaction rate constant of 0.139 min^−1^, where the rate constants for the systems with alternative amines were in the range of 0.070 to 0.143 min^−1^. This demonstrates indeed that the use of amines promotes the primary silanization reaction 1.8 to 3.7 times.

**Figure 7:**
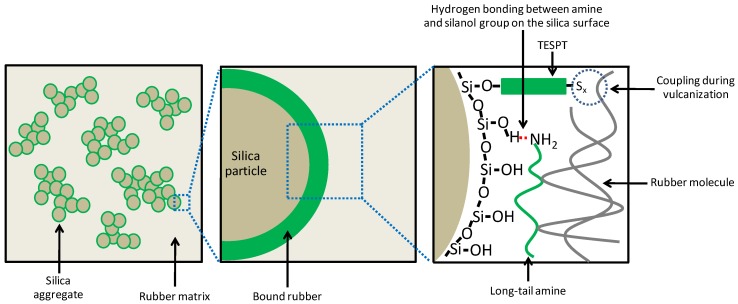
Adsorption of linear aliphatic amine on the silica surface.

The different amine types with similar pKa values but different structures showed variation in the rate constant of the primary silanization reaction, as shown in Figure 6. The amines with linear aliphatic chains (i.e., HEX, DEC, and OCT) showed higher rate constants compared with the amines with a cyclic structure (i.e., CYC, DIC, and QUI). Of the primary amines with linear alkyl chains with the best catalytic effect, HEX (with the shortest alkyl chain) gave the highest rate constant of 0.143 min^−1^. Longer alkyl chains of the aliphatic amines apparently decreased the reaction rate and the presence of a cyclic aliphatic structure further reduced the rate. The bi-cyclic structure of DIC resulted in the lowest rate constant. The difference in promoting the silanization by amines having similar pKa values in the silica/silane system may be attributed to their different adsorption efficiency of the amine molecules on the silica surface. The cyclic ring hampered the adsorption on the silica surface due to steric hindrance, while straight alkyl-chains allowed for easy access to the silanol groups on the silica surface. The increase of one cyclic ring in CYC, to two cyclic rings in DIC, drastically reduced the primary silanization reaction rate by 39%, whereas the increase of the linear aliphatic chain length from C6 to C10 and C18, decreased the reaction rate constants by only about 18% and 20%, respectively. The adsorption of amines with the long aliphatic chains from C10 to C18 on the silica surface introduced slightly more steric effects that may interfere with the silanization reaction. When compared with the short chain amine, the long tail of the amines could shield the silica surface and deactivate the free silanol groups, as displayed in Figure 7.

The secondary silanization reaction that takes place between the left-over alkoxy groups in adjacent silane molecules bound on the silica surface had a much lower rate constant compared to the primary reaction. The data are in agreement with the rate constants reported by Görl et al. [14] for the silica/TESPT system in an *n*-decane model reaction system, in which at 140 °C the primary vs. secondary reaction rate constants were found as 0.122 vs. 0.008 min^−1^, respectively. The rate constants of the secondary silanization reaction of the silica/silane mixture is also shown in Figure 6, in which all mixtures with amines show higher rate constants than the mix without amine. However, the different amine structures had only a small influence on the rate constant of this secondary silanization reaction, found in the range of 0.01 to 0.02 min^−1^. This very low or almost negligible secondary reaction may be attributed to the limited number of silanes grafted on adjacent isolated silanol sites, as a minimum distance between the two SiOH groups of greater than 0.4 nm is estimated in order to be accessible for the incoming silane molecules [16].

**Figure 8:**
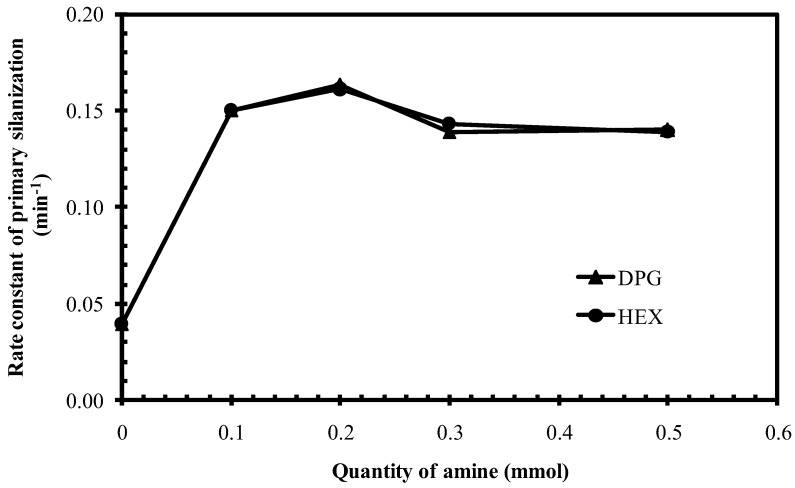
Rate constants of the primary silanization reaction of silica/silane mixtures with different quantities of DPG and HEX.

As displayed in Figure 6, among the amine types studied, HEX gave the highest rate constant of the primary silanization reaction, slightly higher than that of the reference mixture of silica/silane with DPG. Hexylamine is a primary amine with C6 aliphatic tail, while DPG is a secondary amine with aromatic rings, but containing 3 polar -NH groups. These two types of amines were further studied for the effect of the amount on the primary silanization reaction rate constant (Figure 8).

Both DPG and HEX showed the same trend of changes in the rate constants of the primary silanization reaction against the molar quantities used in the model system. With increasing amine loading till 0.2 mmol, the rate constant increased, and thereafter decreased slightly, and tended to level off at an excessive amine loading, as shown in Figure 8. The presence of amine in the silica/silane model system promoted the rate constant of the primary silanization reaction about three fold. The decrease of the silanization reaction rate constant with increasing amounts of amines to more than 0.2 mmol was due to amine adsorption on the silica surface via hydrogen bonding between the amino groups in the amine molecule and the silanol groups on the silica surface causing reduced accessibility for the silane molecules. 

### 3.3. Filler-Elastomer Interactions and Interfacial Compatibility as Enhanced by the Silanization Reaction in Practical Silica-Reinforced NR Compounds

**Figure 9:**
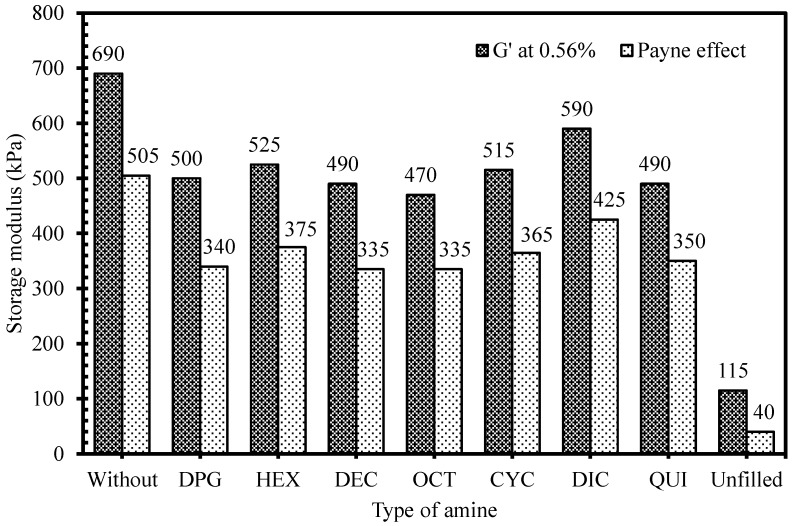
Storage modulus at 0.56% strain and Payne effects of silica-reinforced NR compounds with different amine types.

The strong silica-elastomer interaction in silica/silane-reinforced rubber compounds was obtained via chemical bonding by silane bridge formation. By the efficient use of silane in the silica-reinforced rubber compounds, filler-elastomer interaction was enhanced, and filler-filler interaction could be suppressed. The level of storage modulus at low strain of the filled-rubber compounds indicates stiffness that was raised by the structures of both filler- and rubber-networks. There are four contributions to the storage modulus of the filled rubber compounds: rubber network, hydrodynamic effect, filler-elastomer interaction, and filler-filler interaction. Only the latter was strongly strain-dependent in dependence of the degree of filler-filler interaction. To evaluate the extent of filler-filler interaction or filler networking, the difference in storage modulus at low and high strains, the so-called Payne effect [10] of the uncured silica-reinforced rubber compounds tested under dynamic conditions, could be determined. A decrease of storage modulus with increasing strain for silica-reinforced rubber compounds was due to filler network breakdown, weakening of hydrogen bonding between adjacent silica aggregates, and slip of the rubber chains on the filler surface. As discussed previously for the role of amines in the silica-reinforced rubber compounds, by a combination of an enhanced silanization reaction, deactivation of free silanol groups left over after the silanization and shielding by the aliphatic chains of the amines, a lower storage modulus at low strain and Payne effect was achieved in the amine-containing compounds, when compared to the one without, as shown in Figure 9. The rubber compounds with DEC, OCT, and QUI showed similar levels of the filler-filler interactions compared with the mix with DPG. Among the different amine types, the rubber compound with DIC had the lowest rate constant of the primary silanization reaction (Figure 6), and clearly showed the highest storage modulus and Payne effect (Figure 9). The steric hindrance introduced by the two cyclic rings of DIC hinders the silane molecules to react towards the silica surface, and gives low accessibility towards the left-over free silanol groups after the silanization reaction. For linear aliphatic amines, the levels of filler-filler interactions of the silica-reinforced NR compounds tended to decrease with increasing chain length, because of enhanced hydrophobicity of the silica surface. In order to emphasize the fact that the Payne effect here was strongly related to the filler-filler network, the storage modulus at 0.56% strain and the G’(0.56%)–G’(100%) for a formulation without any filler present, has also been included in Figure 9. The absence of filler clearly lowers the G’(0.56%) tremendously relative to the filled compounds, and the G’(0.56%)–G’(100%) of 40 kPa was due to neo-Hookean behavior of the elastomer per se, and was minimal compared to the Payne effects observed for the filled compounds. 

**Figure 10:**
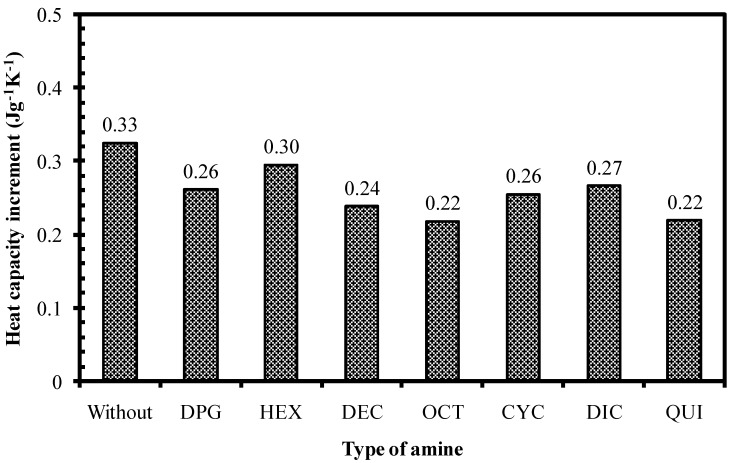
Heat capacity increment of silica-reinforced NR compounds with different amine types.

Another indicator that can be applied to verify the extent of the filler-elastomer interactions in the present work was the change of heat capacity within the rubber compounds as determined by the DSC technique. As the thermal properties of silica-reinforced rubber compounds change with different silica-elastomer interactions, the heat capacity increment (Δ*C*_p_) at *T*_g_, which is related to the fraction of elastomer molecules that change in mobility [12,13], can be used to evaluate the interfacial interaction between filler and elastomer. The elastomer chains on the filler surface that cannot move are often called the immobilized polymer layer, indicating the interfacial compatibility of the silica and rubber phases. The Δ*C*_p_ values of silica-reinforced NR compounds with different amine types are shown in Figure 10. The use of all amines in the silica-reinforced rubber compounds gives lower Δ*C*_p_ than the compound without amine, confirming the enhanced interfacial interactions by the various roles of amines, as previously discussed. The rubber compound with OCT, which showed the lowest Payne effect, exhibited the lowest Δ*C*_p_ when compared to the other compounds. The Δ*C*_p_ values further tended to decrease with increasing length of the alkyl chains, as observed by a reduction of Δ*C*_p_ in the rubber compounds with HEX, DEC, and OCT, respectively. This evidence supports the concept that the tails of the amines could cover the silica surface to enhance hydrophobicity and thus promote silica-elastomer compatibility. In the case of the QUI-containing rubber compound, because of its highest p*K*a value that could also enhance pre-mature coupling between silane and elastomer in addition to the silanization reaction, the immobilized layer of elastomer molecules on the silica surface was increased, and thus, reduced the Δ*C*_p_.

**Figure 11:**
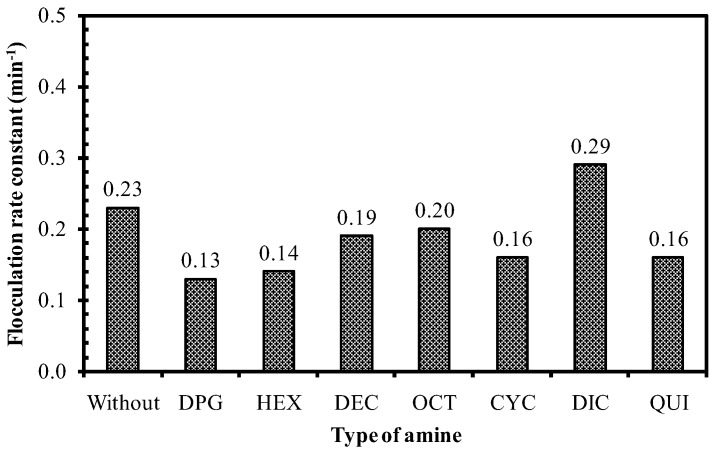
Flocculation rate constant of silica-reinforced NR compounds with different amine types.

For the silica-reinforced rubber compounds, the dispersed silica aggregates in the rubber matrix can again re-agglomerate under heat treatment due to the highly polar functional groups on the silica surface. This phenomenon is so-called flocculation. The flocculation rate constant, which indicates how fast re-agglomeration of silica will take place in the rubber matrix [11] in the beginning phase or scorch period prior to vulcanization of the silica-reinforced NR compounds with different amine types, is shown in Figure 11. The presence of amines in the silica/silane system, except for the use of DIC, resulted in a lower flocculation rate constant compared to the compound without amine. Diphenyl guanidine and HEX that gave similar rate constants of the primary silanization reaction in the model systems exhibited the same flocculation rate constants as a result of the enhanced silanization by the amines. Among the linear aliphatic amines, the rubber compound with HEX showed a lower flocculation rate constant than with DEC and OCT. The compounds containing the latter two amines with longer alkyl chains could flocculate faster compared to HEX, which gave a greater extent of the silanization reaction. The physical interactions were diminished at elevated temperature, while the chemical interactions remained. Flocculation of dispersed silica in the rubber matrix could still take place after the disappearance of the silica-amine interaction during thermal treatment. The amine with the lowest primary silanization reaction rate constant (i.e., DIC with two steric cyclic rings) (Figure 6), displayed the highest flocculation rate constant (Figure 11) in correspondence with its highest Payne effect (Figure 9). The results demonstrate that the study on the silica/silane model systems and the practical silica-reinforced rubber compounds compared well with each other. 

## 4. Conclusions

The kinetics of the silanization reaction in the silica/silane model systems containing various amine types reveal that such amines with different chemical structures provide quite diverse influences on the rate constant of the primary silanization reaction, but almost do not change the rate constant of the secondary silanization reaction. The presence of all amine types in the silica/silane systems increases the rate constant of the primary silanization reaction compared to the system without amine. The primary silanization reaction rate constants in model systems with DPG and HEX reach an optimum when 0.2 mmol of amines was used. The amine-catalyzed silanization reaction occurs after the amine is adsorbed on the silica surface via hydrogen bonding between NH-groups of the amine molecules and silanol groups on the silica surface. The linear aliphatic amines with longer alkyl-chains promote hydrophobicity by a shielding effect, but hinder the reactive silanol-groups on the silica surface, as observed in a reduction of the rate constant of the primary silanization reaction and lower Payne effect. The steric cyclic aliphatic structure of CYC, DIC, and QUI resulted in a lower rate constant of the primary silanization reaction in the model silica/silane system, compared to the linear aliphatic amines (i.e., HEX, DEC, and OCT). With regard to practical silica-reinforced rubber compounds, the properties of uncured compounds are influenced by both filler-elastomer interaction promoted by the silanization reaction and physical adsorption of amines on the silica surface. The amine types that give a higher primary silanization reaction rate constant show a lower flocculation rate constant in the practical compounds. For the systems with a bit lower primary silanization reaction rate constant, but higher extent of shielding or physical adsorption, the rubber compounds show lower Payne effect but still higher flocculation rate constant due to the physical shielding. Compared with DPG, linear aliphatic amines can enhance the silanization reaction and yield properties of the silica-reinforced rubber compounds to match those of DPG. The cyclic aliphatic amines show inferior kinetics parameters in the silica/silane model system and poorer properties of uncured silica-rubber compounds compared to linear aliphatic amines, but are clearly better than the system without amine.

## Figures and Tables

**Table 1:** Typical information of amines used in this study.

Chemical Name	Code	Structure	pKa	MW (g/mol)
Diphenyl guanidine	DPG	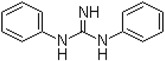	10.10	211.3
Hexylamine	HEX	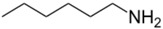	10.56	101.2
Decylamine	DEC	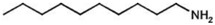	10.64	157.3
Octadecylamine	OCT	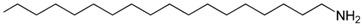	10.60	269.5
Cyclohexylamine	CYC	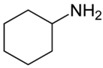	10.64	99
Dicyclohexylamine	DIC	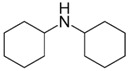	10.40	181
Quinuclidine	QUI	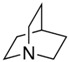	11.50	111

**Table 2:** Gas chromatography (GC) chromatographic conditions.

Items	Conditions
Column type	BPX5
Length of column	15 m
Internal diameter	0.32 mm
Film thickness	0.25 μm
Detector	Flame ionization detector (FID)
Flow rate	2 mL/min
Temperature	250 °C

**Table 3:** Rubber compound formulation.

Ingredients	Quantity (phr)
NR	100
Silica	55
TESPT	5
ZnO	3
Stearic acid	1
TMQ	1
TDAE oil	8
DPG *	0.55

(*) Other types of amines were used based on molar equivalents to 0.55 phr of DPG. NR = natural rubber; TESPT = bis(3-triethoxysilylpropyl)-tetrasulfide; TMQ = 2,2,4-triMethyl-1,2-dihydroQuinoline; and TDAE = treated distillate aromatic extract.

**Table 4:** Mixing procedure.

Cumulative Mixing Time (min)	Step of Mixing
0	NR
2	½ Silica + ½ TESPT + ½ amine
7	½ Silica + ½ TESPT + ½ amine + TDAE oil
12	ZnO + Stearic acid + TMQ
15	Discharge

## References

[1] Rauline R. (1992). (to Michelin and Cie). Eur. Pat. Patent.

[2] Reuvekamp L.A.E.M., ten Brinke J.W., van Swaaij P.J., Noordermeer J.W.M. (2002). Effects of mixing condition on the reaction of TESPT silane coupling agent during mixing with silica filler and tire rubber. Kautsch. Gummi Kunstst..

[3] Kaewsakul W., Sahakaro K., Dierkes W.K., Noordermeer J.W.M. (2012). Optimization of mixing conditions for silica-reinforced natural rubber tire tread compounds. Rubber Chem. Technol..

[4] Kaewsakul W., Sahakaro K., Dierkes W.K., Noordermeer J.W.M. (2013). Optimization of rubber formulation for silica-reinforced natural rubber compounds. Rubber Chem. Technol..

[5] Kaewsakul W., Sahakaro K., Dierkes W.K., Noordermeer J.W.M. (2015). Mechanistic aspects of silane coupling agents with different functionalities on reinforcement of silica-filled natural rubber compounds. Polym. Eng. Sci..

[6] Mihara S., Datta R.N., Talma A.G., Noordermeer J.W.M. (2011). Rubber Composition. U.S. Patent.

[7] Hayichelaeh C., Reuvekamp L.A.E.M., Blume A., Noordermeer J.W.M., Sahakaro K. (2017). Reinforcement of natural rubber by silica/silane in dependence of different amine types. Rubber Chem. Technol..

[8] (2002). Agency for Toxic Substances and Disease Registry. http://www.atsdr.cdc.gov/toxfaqs/tfacts171.pdf.

[9] Wolff S., Wang M.J., Tan E.H. (1993). Filler-elastomer interactions. part vii. study on bound rubber. Rubber Chem. Technol..

[10] Payne A.R. (1966). Effect of dispersion on dynamic properties of filler-loaded rubbers. Rubber Chem. Technol..

[11] Mihara S., Datta R.N., Noordermeer J.W.M. (2009). Flocculation in silica reinforced rubber compounds. Rubber Chem. Technol..

[12] Zhong B., Jia Z., Hu D., Luo Y., Jia D. (2015). Reinforcement and reinforcing mechanism of styrene–butadiene rubber by antioxidant-modified silica. Compos. Part A.

[13] Fragiadakis D., Bokobza L., Pissis P. (2011). Dynamics near the filler surface in natural rubber-silica nanocomposites. Polymer.

[14] Görl U., Hunsche A., Mueller A., Koban H.G. (1997). Investigations into the Silica/Silane Reaction System. Rubber Chem. Technol..

[15] Blume A., Thibault-Starzyk F. (2017). Deciphering the silica/silane reaction mechanism for the development of a new generation of low rolling resistance tires: Part 1—Characterization by in situ IR spectroscopy. Rubber Fibres Plast. Int..

[16] Blume A., Thibault-Starzyk F. (2017). Deciphering the silica/silane reaction mechanism for the development of a new generation of low rolling resistance tires: Part 2—Transfer of results from model examinations into practice. Rubber Fibres Plast. Int..

[17] Blume A. (2011). Kinetics of the silica-silane reaction. Kautsch. Gummi Kunstst..

[18] Mihara S. (2009). Reactive Processing of Silica-Reinforced Tire Rubber. Ph.D. Thesis.

[19] Li H.-L., Fu A.-P., Xu D.-S., Guo G.-L., Gui L.-L., Tang Y.-Q. (2002). In situ silanization reaction on the surface of freshly prepared porous silicon. Langmuir.

[20] Kim K.-J., VanderKooi J. (2005). Moisture effects on tespd-silica/cb/sbr compounds. Rubber Chem. Technol..

[21] Tripp C.P., Hair M.L. (1993). Chemical attachment of chlorosilanes to silica: A two-step amine-promoted reaction. J. Phys. Chem..

[22] White L.D., Tripp C.P. (2000). Reaction of (3-aminopropyl)dimethylethoxysilane with amine catalysts on silica surfaces. J. Colloid Interface Sci..

[23] Kanan S.M., Tze W.T.Y., Tripp C.P. (2002). Method to double the surface concentration and control the orientation of adsorbed (3-aminopropyl)dimethylethoxysilane on silica powders and glass slides. Langmuir.

